# Rapid conjugation of antibodies to toxins to select candidates for the development of anticancer Antibody-Drug Conjugates (ADCs)

**DOI:** 10.1038/s41598-020-65860-x

**Published:** 2020-06-01

**Authors:** Ricarda M. Hoffmann, Silvia Mele, Anthony Cheung, Daniel Larcombe-Young, Gintare Bucaite, Eirini Sachouli, Iva Zlatareva, Hassan O. J. Morad, Rebecca Marlow, James M. McDonnell, Mariangela Figini, Katie E. Lacy, Andrew J. N. Tutt, James F. Spicer, David E. Thurston, Sophia N. Karagiannis, Silvia Crescioli

**Affiliations:** 1 St. John’s Institute of Dermatology, School of Basic & Medical Biosciences, King’s College London, Tower Wing, 9th Floor, Guy’s Hospital, London, SE1 9RT United Kingdom; 2 NIHR Biomedical Research Centre at Guy’s and St. Thomas’s Hospitals and King’s College London, King’s College London, London, United Kingdom; 3 Breast Cancer Now Research Unit, School of Cancer & Pharmaceutical Sciences, King’s College London, Guy’s Cancer Centre, London, United Kingdom; 4 Breast Cancer Now Toby Robins Research Centre, Institute of Cancer Research, London, United Kingdom; 5 Institute of Pharmaceutical Science, School of Cancer and Pharmaceutical Sciences, King’s College London, London, SE1 9NH United Kingdom; 6 Femtogenix Ltd, Lawes Open Innovation Hub, Rothamsted Research, West Common, Harpenden, Hertfordshire AL5 2JQ United Kingdom; 7 Randall Centre for Cell and Molecular Biophysics, King’s College London, London, SE1 1UL United Kingdom; 8 Asthma UK Centre in Allergic Mechanisms of Asthma, King’s College London, London, SE1 1UL United Kingdom; 9 Biomarker Unit, Department of Applied Research and Technology Development, Fondazione, IRCCS Istituto Nazionale dei Tumouri Milano, 20133 Milan, Italy; 10 School of Cancer & Pharmaceutical Sciences, King’s College London, 3rd Floor, Guy’s Hospital, London, United Kingdom

**Keywords:** Cancer, Tumour immunology, Drug screening, Immunotherapy, Tumour immunology

## Abstract

Antibody-Drug Conjugates (ADCs) developed as a targeted treatment approach to deliver toxins directly to cancer cells are one of the fastest growing classes of oncology therapeutics, with eight ADCs and two immunotoxins approved for clinical use. However, selection of an optimum target and payload combination, to achieve maximal therapeutic efficacy without excessive toxicity, presents a significant challenge. We have developed a platform to facilitate rapid and cost-effective screening of antibody and toxin combinations for activity and safety, based on streptavidin-biotin conjugation. For antibody selection, we evaluated internalization by target cells using streptavidin-linked antibodies conjugated to biotinylated saporin, a toxin unable to cross cell membranes. For payload selection, we biotinylated toxins and conjugated them to antibodies linked to streptavidin to evaluate antitumour activity and pre-clinical safety. As proof of principle, we compared trastuzumab conjugated to emtansine via streptavidin-biotin (Trastuzumab-SB-DM1) to the clinically approved trastuzumab emtansine (T-DM1). We showed comparable potency in reduction of breast cancer cell survival *in vitro* and in growth restriction of orthotopic breast cancer xenografts *in vivo*. Our findings indicate efficient generation of functionally active ADCs. This approach can facilitate the study of antibody and payload combinations for selection of promising candidates for future ADC development.

## Introduction

Antibody-Drug Conjugates (ADCs) combine the high specificity of antibodies for their target antigens with the cytotoxic effects of payloads to more specifically guide toxic agents towards cancer cells^1^. The field of ADCs has been undergoing significant expansion since the first agent gemtuzumab ozogamicin (Mylotarg), targeting CD20-expressing B cell lymphomas, was approved by the Food and Drug Administration (FDA) in 2000^2^. However, despite the potential for this therapeutic modality and the launch of many clinical studies, attrition rates are high due to several challenges such as low efficacy and “on-target” toxicity on normal cells. Until now, only eight ADCs along with two immunotoxins have been approved by the regulatory agencies^1^. In order to improve the success rate and wider applicability of ADCs in oncology there is a need to rapidly generate and test multiple targets and antibody-payload combinations. This could be expedited by the development of efficient screening tools to facilitate the generation and evaluation of different antibody-toxin pairings, a process that is presently not efficient due to the variety of linking strategies in use. To facilitate the screening of larger libraries of payloads which can be lengthy, technically-challenging, costly and time-consuming^3^, we have developed a platform that allows rapid and cost-effective selection of targets, antibodies and payloads suitable for ADC development.

For ADC development, specificity for the target antigen and internalization of the antibody are key mechanisms for targeted drug delivery to cells. Therefore, the main focus during antibody screening for ADC development is placed on the Fab-region of the antibody^4^, to search for internalizing antibodies/target antigen complexes. Different technologies have been employed such as antibody labelling with fluorescent or pH sensitive dyes for confocal microscopy or flow cytometric analysis^5^, or Saporin-based reporter assays. For the latter, biotinylated antibody is conjugated to streptavidin linked to Saporin, a potent 30 kDa ribosome-inactivating plant protein unable to pass the plasma membrane but toxic if internalized. Therefore antibody-Saporin complex internalization can be indirectly evaluated by detection of target cell viability^6,7^.

Although Streptavidin-Biotin linking has not been extensively explored in the context of generating ADCs, this system has been a popular approach in other *in vitro* and *in vivo* applications in cell biology and proteomics, as well as drug labelling and delivery^8,9^. The technology owes its popularity to the extraordinarily high affinity between Streptavidin and Biotin, creating one of the strongest non-covalent interactions known in biology which has become useful in many applications^10^.

Here, we report the rapid conjugation and generation of antibody-drug combinations to facilitate early antibody and payload assembly, pre-clinical potency, functional evaluation and selection of promising antibody-drug pairs for future ADC development. This methodology relies on antibody streptavidin labelling and conjugation to biotinylated payloads. Toxic small molecules used for ADC development must be amenable to conjugation^11^ through specific linkers and so must contain suitable chemically-reactive functional groups^12^. Our approach takes advantage of functional groups such as amines or thiols for payload biotinylation followed by conjugation to streptavidin-linked antibodies to rapidly generate Antibody-Streptavidin-Biotin-Drug (Antibody-SB-Drug) conjugates. To assess antibody internalization, we have evaluated cell viability using ADCs generated by conjugating streptavidin-linked antibodies of interest to biotinylated Saporin. As a proof of concept, we generated a trastuzumab-Streptavidin-Biotin-DM1 conjugate (Trastuzumab-SB-DM1) which we evaluated alongside the clinically approved ADC trastuzumab-DM1 (T-DM1, Kadcyla) in both *in vitro* and *in vivo* experiments. This process is designed to facilitate evaluation of antibody and payload combinations for the further development of promising ADC candidates prepared by conventional homogenous linking approaches.

## Results

### Streptavidin-Biotin linking for Saporin-based ADC generation to investigate antibody internalization

**Figure 1:**
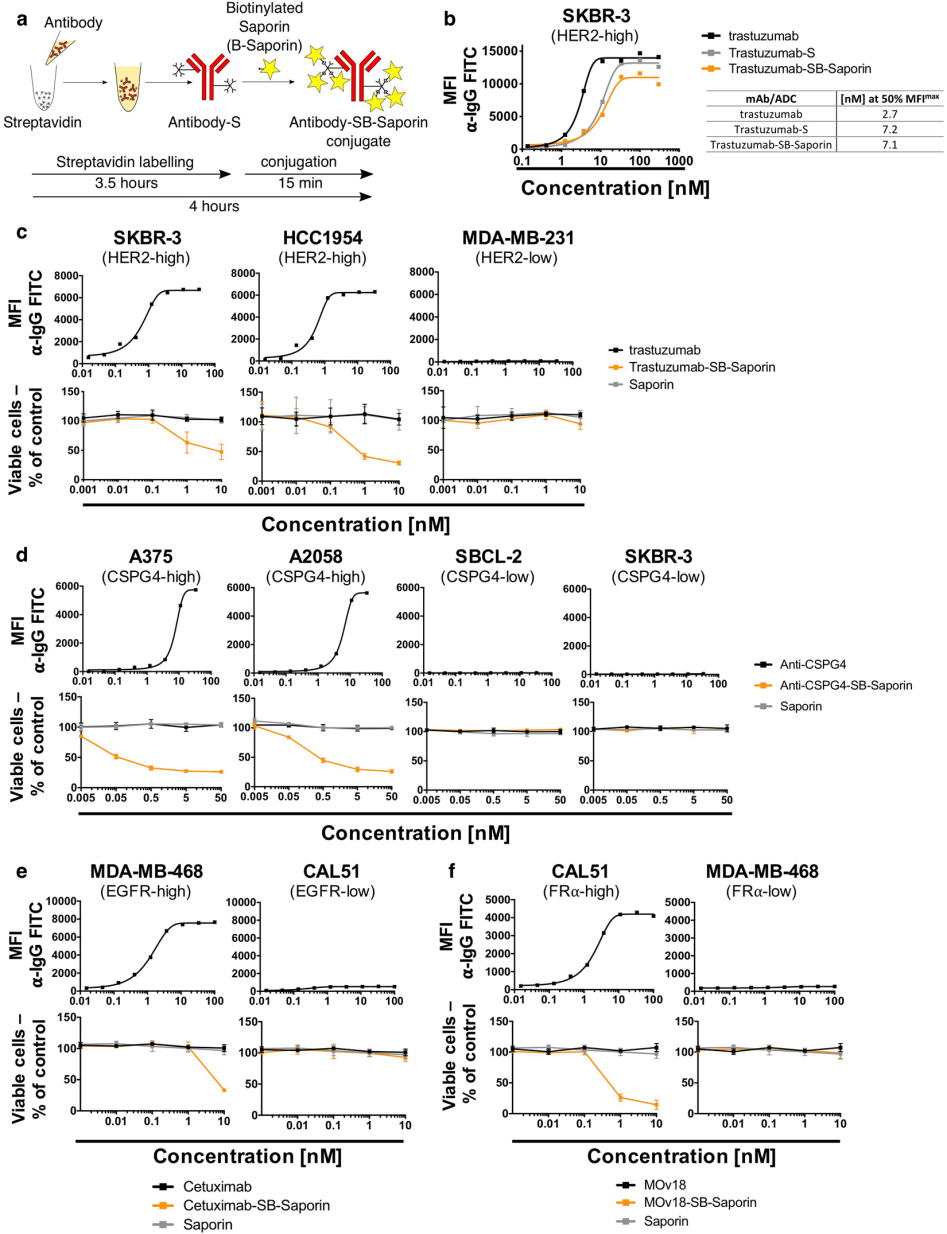
Antibody Streptavidin linked to Biotin-Saporin as payload can be used to investigate antibody internalization (**a**) Schematic of Antibody-SB-Saporin conjugate generation. Streptavidin is conjugated randomly to lysine residues of the antibody with an average of two molecules of streptavidin per antibody. Streptavidin-linked antibody was conjugated to Monobiotin-Saporin at a molar ratio of 1:6. (**b**) Binding assay of trastuzumab, Trastuzumab-SB-Saporin and Trastuzumab-S to Her2-high SKBR-3 breast cancer cells and their concentrations at 50% of the maximum mean fluorescence intensity. (**c–f)** Top panels, target antigen recognition by naked antibodies, anti-Her2 trastuzumab (**c**), anti-CSPG4 (**d**), anti-EGFR Cetuximab (**e**), anti-FRα MOv18 (**f**) on cancer cell lines expressing different target antigens expression (Her2 (**c**), CSPG4 (**d**), EGFR (**e**), FRα (**f**)). Bottom panels (**c–f**), investigation of cell viability upon treatment with the naked antibody (black), antibody-Streptavidin-Biotin-Saporin conjugate (orange) or Saporin alone (grey). The ribosome inhibitor Saporin, unable to enter the cell alone, can be used to investigate antibody internalization by measuring viability (MTS) of antibody-SB-Saporin conjugate-treated cells. N = 1 for all binding assays and N = 3-4 independent experiments for MTS studies. All experiments were performed in triplicate, error bars represent Standard Deviation (SD).

To accomplish selective cytotoxicity, ADCs need to enter the target cell upon recognizing an antigen in order to expose the cell to the toxin. We aimed to develop a rapid and simple method to investigate the propensity of an antibody to internalize in order to select suitable antibodies for ADC development. For this we employed rapid Streptavidin-Biotin linking to conjugate the antibody of interest to Saporin (Fig. 1a), a 30 kDa ribosome-inhibitor protein unable to enter the cell alone and therefore only toxic to the cell when conjugated to an internalizing antibody. We rapidly produced Saporin-based ADCs (Antibody-SB-Saporin) using a two-step approach: antibody labelling with streptavidin (Antibody-S) (3.5 hours) followed by conjugation to biotinylated-Saporin (B-Saporin) (15 min). Trastuzumab has 41 lysine residues available for random conjugation, which can potentially result in the binding of streptavidin to both the Fab and Fc regions (Supplementary Fig. 1). To conjugate the antibody to streptavidin we used a commercially available kit which, according to the manufacturer, allows the conjugation of an average of two molecules of streptavidin per antibody.

We first evaluated whether streptavidin labelling of the antibody and conjugation to Biotin-Saporin impaired the Her2 antigen binding capacity of the monoclonal antibody trastuzumab on Her2-high SKBR-3 breast cancer cells. We showed that trastuzumab-streptavidin (Trastuzumab-S) and Trastuzumab-SB-Saporin conjugated with a molar ratio of 1:6 retained the ability to bind Her2. However, higher concentrations of antibody-conjugates were required to reach 50% of the maximal mean fluorescence intensity (MFI^max^) compared to unconjugated trastuzumab ([nM] at 50% MFI^max^ 7.2 and 7.1 for Trastuzumab-S and Trastuzumab-SB-Saporin respectively and 2.7 for trastuzumab) (Fig. 1b, Supplementary Fig. 1). We evaluated the internalization propensities of four antibodies of different specificities in target cell lines with high- and low expression of target antigen. Target antigen recognition was examined by flow cytometry (Fig. 1c–f, top panels) and antibody internalization was indirectly measured by analysing cell viability after treatment with Saporin-based ADCs, naked antibody or Saporin alone (Fig. 1c–f, bottom panels). Trastuzumab-SB-Saporin triggered a concentration dependent decrease in cell viability in the Her2-high SKBR-3 and HCC1954 breast cancer cells (Fig. 1c). We investigated if the molar ratio of Saporin conjugated per antibody could impact antigen binding and tumour cell viability. Using different molar ratios (1:2, 1:4, 1:6,1:8) to conjugate Trastuzumab-Streptavidin and biotinylated-Saporin, we showed decreased binding to Her2-expressing cancer cells and reduced cancer cell viability with higher molar ratios of Trastuzumab-SB-Saporin conjugation. As anticipated, Trastuzumab-SB-Saporin had no impact on the viability of Her2-low MDA-MB-231 basal breast cancer cells (Fig. 1c) or on other Her2-low cell lines or on monocytic cell lines expressing Fcγ receptors (Supplementary Fig. 2). Anti-CSPG4-SB-Saporin, specific for the melanoma associated antigen chondroitin sulfate proteoglycan 4 (CSPG4), caused a concentration-dependent decrease in cell viability of CSPG4-high A375 and A2058 melanoma cell lines, but had no toxic effects on the CSPG4-low SBCL-2 and SKBR-3 breast cancer cells (Fig. 1d). Cetuximab-SB-Saporin, targeting the human epidermal growth factor receptor (EGFR), caused a concentration-dependent decrease in cell viability of EGFR-high MDA-MB-468, but not of EGFR-low CAL51, breast cancer cells (Fig. 1e). MOv18-SB-Saporin, targeting the tumour-associated antigen Folate Receptor α (FRα) caused a concentration-dependent decrease in viability of FRα-high CAL51, but not of FRα-low MDA-MB-468 breast cancer cells (Fig. 1f). As expected, none of the cell lines studied showed any loss in cell viability when treated with Saporin alone or naked antibodies (Fig. 1c–f, bottom panels), since concentrations of naked antibodies were well-below those required for these agents to potentiate direct effects on tumour cells.

Therefore, antibody streptavidin labelling followed by conjugation to biotinylated Saporin can help generate Antibody-SB-Saporin conjugates in less than 4 hours. These can be used to investigate the antibody’s ability to internalize, pivotal for ADC development, here demonstrated using two clinically available and two experimental recombinant antibodies.

### ADCs generated by Streptavidin-Biotin conjugation are functionally active *in vitro*

We sought to investigate whether Streptavidin-Biotin conjugation may be suitable for the conjugation of small molecule payloads typically used for ADC development.

**Figure 2:**
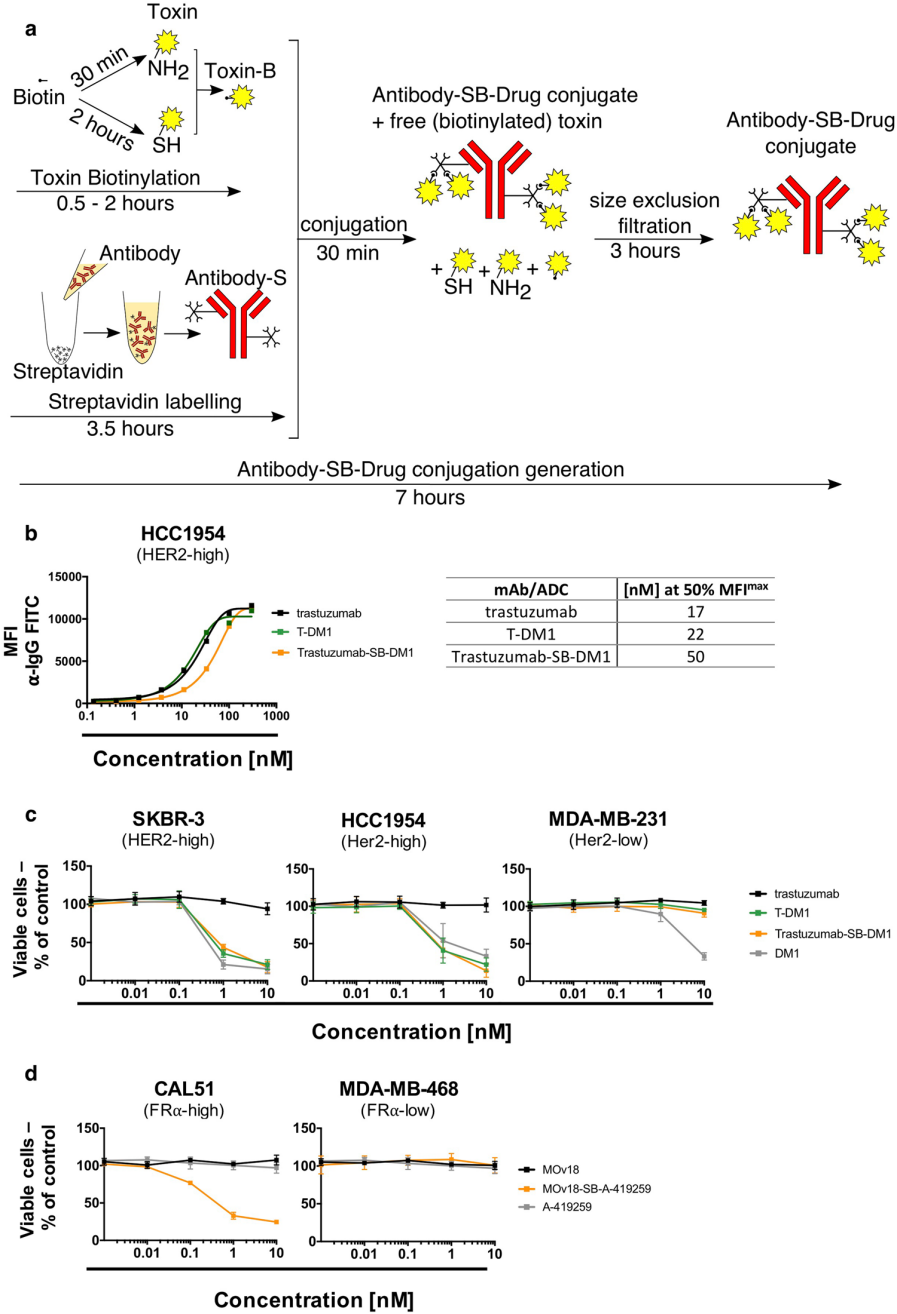
Antibody-drug conjugate generation via Streptavidin-Biotin linking results in functionally active ADCs with anti-tumour potency *in vitro* (**a**) Schematic of Antibody-SB-Drug conjugate generation. Toxins containing functional groups such as thiols or amines can be directly biotinylated using commercially available kits and conjugated to streptavidin-linked antibodies for rapid Antibody-SB-Drug production. (**b**) Binding assay of trastuzumab, T-DM1 and Trastuzumab-SB-DM1 (molar ratio 1:3.5) to Her2-high HCC1954 breast cancer cells and their concentrations required to reach 50% of the maximum mean fluorescence intensity (MFI^max^). (**c**) Investigation of cell viability of breast cancer cell lines with high (SKBR-3, HCC1954) and low (MDA-MB-231) Her2 expression. Tumour cells were treated with the naked antibody Trastuzumab, positive control T-DM1, trastuzumab-Streptavidin-Biotin-DM1 (Trastuzumab-SB-DM1) or DM1 toxin alone. (**d**) Investigation of cell viability of breast cancer cell lines with high (CAL51) and low (MDA-MB-468) Folate Receptor α expression upon treatment with the naked antibody MOv18, MOv18-Streptavidin-Biotin-A-419259 (molar ratio 1:8) (MOv18-SB-A-419259) or Src-inhibitor A-419259 alone. N = 3-4 independent experiments for all MTS studies, each condition performed in triplicate, error bars represent Standard Deviation (SD).

Toxins for ADC development often contain reactive groups such as amines or thiols that are used for conjugation to antibodies via specific linkers. We evaluated two examples of generating ADCs by Streptavidin-Biotin conjugation with small molecule payloads. We biotinylated two types of toxins presenting primary amine or thiol reactive groups (B-Drug) and we conjugated these to streptavidin-linked antibodies (Antibody-S) to produce Antibody-SB-Drug conjugates in as little as 7 hours (Fig. 2a). We then tested efficacy and safety by investigating tumour and non-malignant cell viability with ADC, naked antibody or payload only treatment.

Firstly, an ADC composed of trastuzumab and DM1 (Trastuzumab-SB-DM1) biotinylated via the thiol group of DM1 was tested in parallel with the clinically used trastuzumab-DM1 (T-DM1) consisting of DM1 and trastuzumab but linked via mcc-maleimide linking^13^. To mimic the drug-antibody ratio (DAR) of the clinically available T-DM1, we conjugated Trastuzumab-SB-DM1 using a molar ratio of 3.5 molecules of DM1 per one trastuzumab antibody^14^. We demonstrated similar binding kinetics of Trastuzumab-SB-DM1 compared with clinically available naked antibody trastuzumab as well as with T-DM1, with 50% of the maximal mean florescence intensity reached at 50 nM (Trastuzumab-SB-DM1), 17 nM (trastuzumab) and 22 nM (T-DM1) (Fig. 2b). We evaluated the effect of the ADCs on breast cancer cell lines expressing different levels of the Her2 target antigen using the naked antibody trastuzumab and the DM1 toxin alone as controls.

In Her2-high SKBR-3 and HCC1954 breast cancer cells, T-DM1 and Trastuzumab-SB-DM1 showed comparable reduction of cell viability, similar to DM1 alone, while the naked antibody showed no effect on cell viability (Fig. 2c). As controls, in the Her2-low MDA-MB-231 breast cancer cells and in the FcγR expressing U937 monocytic cell line, neither T-DM1 nor Trastuzumab-SB-DM1 had any effects on cell viability, similarly to the naked antibody. As expected, cells treated with the cell-permeable payload DM1 alone showed decreased cell viability in both cell lines (Fig. 2c and Supplementary Fig. 3).

Further, we biotinylated a Src family kinase inhibitor (A-419259) via its primary amine and conjugated it to the streptavidin-linked anti-FRα antibody MOv18 to produce the ADC MOv18-SB-A-419259 with a molar ratio of 8 molecules of A-419259 per one MOv18 antibody to maximise the amount of payload per antibody. This choice was based on the cancer-promoting Erk pathway signalling mechanisms in basal breast and ovarian tumours and on the ability of A-419259 to decrease ERK kinase activity in cancer cells, as we previously reported^15–17^. We then tested effects on the viability of tumour cells expressing high and low levels of FRα. MOv18-SB-A-419259 exerted a concentration-dependent decrease in tumour cell viability in the FRα overexpressing CAL51 basal breast cancer cells while unconjugated MOv18 antibody or payload A-419259 alone had no impact on cell viability (Fig. 2d, left panel). Contrastingly, neither MOv18-SB-A-419259, nor MOv18 or A-419259 had any effects on the viability of low FRα-expressing MDA-MB-468 cancer cells (Fig. 2d, right panel).

Therefore, Streptavidin-Biotin conjugation can facilitate linking antibodies to payloads with functional groups such as thiols or primary amines to generate Antibody-SB-Drug conjugates which can be used to evaluate the complex’s *in vitro* tumour cell-targeting effects.

**Figure 3:**
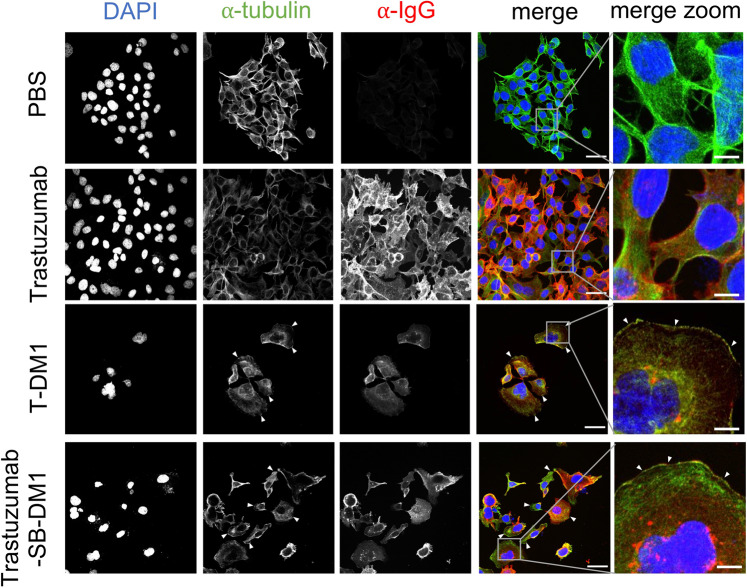
Trastuzumab-SB-DM1 internalization and mechanism of action is comparable to the clinically available T-DM1. HER2-high HCC1954 breast cancer cells were treated with naked antibody trastuzumab, control ADC T-DM1, Trastuzumab-SB-DM1 or PBS for 72 h and were stained for anti-IgG to investigate the internalization of trastuzumab antibody/ADC and anti-β-tubulin to compare tubulin disassembly (white arrowheads) upon treatment with T-DM1 and Trastuzumab-SB-DM1 ADCs. Scale bar, overview 50 μm, zoom 10 μm.

We next investigated whether a Streptavidin-Biotin conjugated ADC can exert comparable mechanisms of tumour cell killing to an ADC produced by a conventional linking method. For this we compared tubulin polymerization disruption in Her2-high HCC1954 cells treated with Trastuzumab-SB-DM1 or with T-DM1. The main mechanism of action of T-DM1 relies on the effects of the toxic payload DM1 which is known to cause microtubule disassembly^18^. We therefore labelled HCC1954 tumour cells treated with PBS, trastuzumab alone, T-DM1 or Trastuzumab-SB-DM1 with anti-IgG to confirm antibody internalization and with anti-β-tubulin and we compared tubulin formation between the two ADCs by confocal microscopy (Fig. 3). Antibody internalization was evident in cells treated with trastuzumab alone, T-DM1 or Trastuzumab-SB-DM1. Microtubule disassembly of cells treated with both ADCs T-DM1 and Trastuzumab-SB-DM1 (Fig. 3, white arrowheads) was observed, while normal tubulin assembly was found in cells treated with PBS or trastuzumab alone.

These results suggest that Streptavidin-Biotin conjugation can retain the mechanism of toxin-mediated tumour cell killing of a conventionally-linked ADC.

### Trastuzumab-SB-DM1 displays tumour-growth restricting potency in vivo comparable to that of the clinically available T-DM1

**Figure 4:**
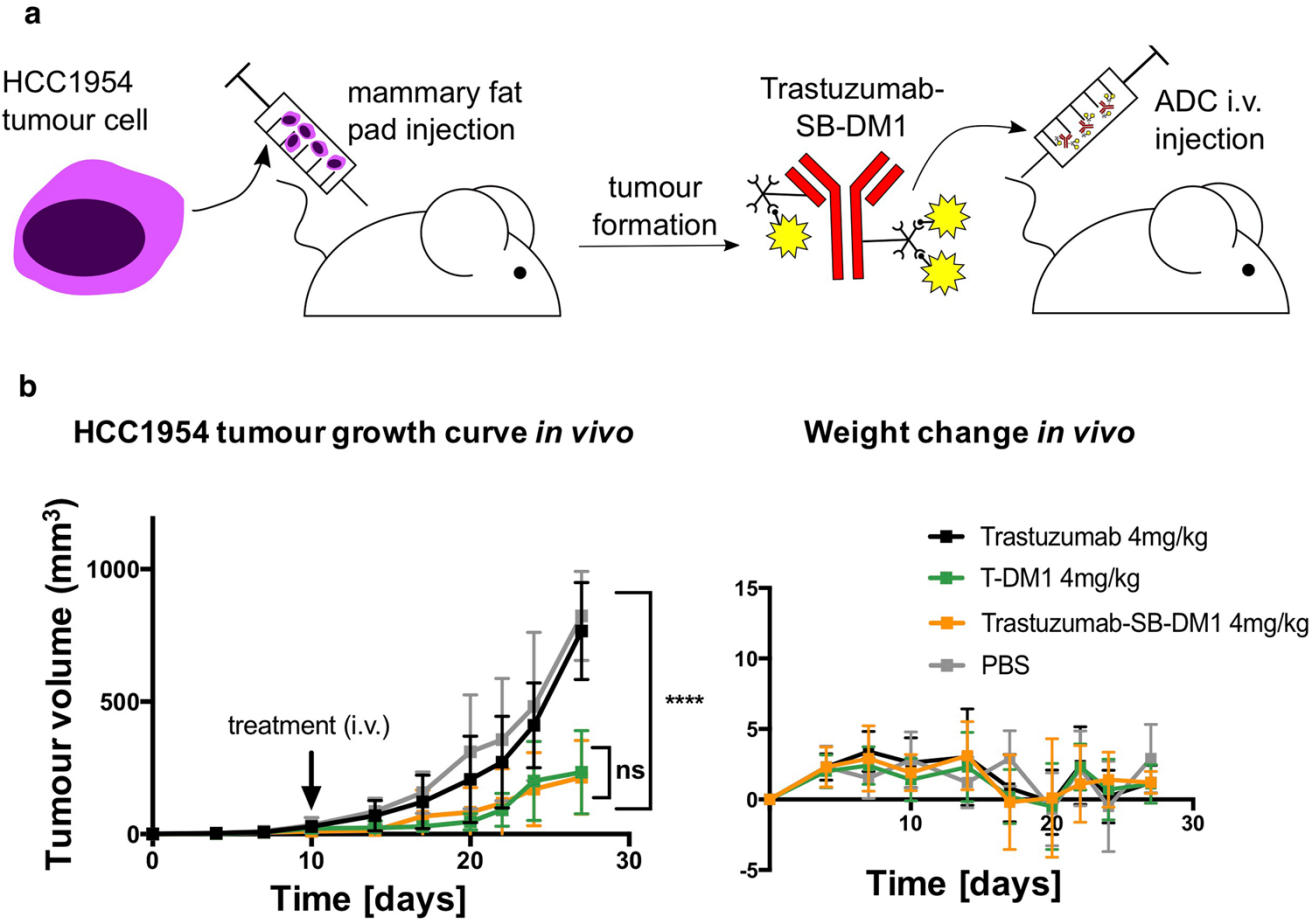
Trastuzumab-SB-DM1 and T-DM1 display comparable effects in restricting orthotopic mammary fat pad breast tumour growth *in vivo* (**a**) Schematic of Trastuzumab-SB-DM1 *in vivo* treatments. Human HCC1954 mammary fat pad xenograft-bearing mice were treated intravenously (i.v.) with one dose of Trastzumab-SB-DM1 following establishment of palpable tumours. (**b**) Growth curves of HCC1954 orthotopic tumours, mean ± SEM (left panel) and mouse weight mean ± SD (right panel). N = 10 mice per treatment group, treated with a single-dose of trastuzumab (4 mg/kg), T-DM1 (4 mg/kg), Trastuzumab-SB-DM1 (4 mg/kg), or PBS (right panel). ****P < 0.0001, by Dunnett’s multiple comparisons test.

To gain insight into the ability of a Streptavidin-Biotin conjugated ADC to restrict tumour growth *in vivo*, we compared Trastuzumab-SB-DM1 and the clinically used T-DM1 in an orthotopic human breast cancer xenograft grown in the mammary fat pads of immunocompromised (NSG) mice (Fig. 4a). HCC1954 xenografts treated with a single 4 mg/kg dose of Trastuzumab-SB-DM1 showed a significant reduction of tumour growth compared to mice given PBS vehicle and naked trastuzumab. Restriction of tumour growth was comparable to that shown with administration of T-DM1 (tumour size at end of study + /- SEM per group in mm^3^: PBS 839 ± 52, trastuzumab 767 ± 58, T-DM1 234 ± 50, Trastuzumab-SB-DM1 214 ± 44) (P < 0.0001, Dunnett’s multiple comparisons test) (Fig. 4b, left panel). As expected a single dose of 4 mg/kg naked antibody was not sufficient to restrict tumour growth compared with vehicle treatment^19^. Treatments had no significant impact on animal weight, suggesting that none of the agents, including Trastuzumab-SB-DM1, exerted any overt toxicities *in vivo* (Fig. 4b, right panel).

In conclusion, we demonstrated that Trastuzumab-SB-DM1 ADC, generated by Streptavidin-Biotin conjugation, showed comparable tumour-growth restricting qualities *in vitro* as well as *in vivo* compared with the clinically available T-DM1, in the absence of systemic toxicities. These data suggest that ADCs generated by Streptavidin-Biotin conjugation may be suitable for *in vivo* pre-clinical screening of antibody-drug ADC combinations.

## Discussion

ADCs have emerged as a promising modality for targeted cancer treatment and their development is one of the fastest growing areas of therapeutics research^20^. Dramatically increased numbers of ADCs in clinical development is evidence for growing interest in the field. This indicates a need to screen antibodies as well as payloads for selection of optimal combinations. Pre-clinical and clinical experience has led to the development of improved ADCs with more stable linkers between the antibodies and toxins to help reduce potential off-target toxic effects in early phase clinical trials^21,22^. However, linking strategies are complex, requiring expertise and well-equipped laboratories, most often conducted by contract research organizations (CROs) focused on linking payloads to antibodies. Therefore, prior to engaging in the financial and time-consuming investment of commissioning ADC conjugation, it is crucial to ascertain that the chosen antibody and payload combination features optimal potential for future ADC development. To address this need, here we established and evaluated a platform that allows rapid linking of payloads to antibodies to produce ADCs for early pre-clinical *in vitro* and *in vivo* studies of targets, antibodies and payload combinations.

**Figure 5:**
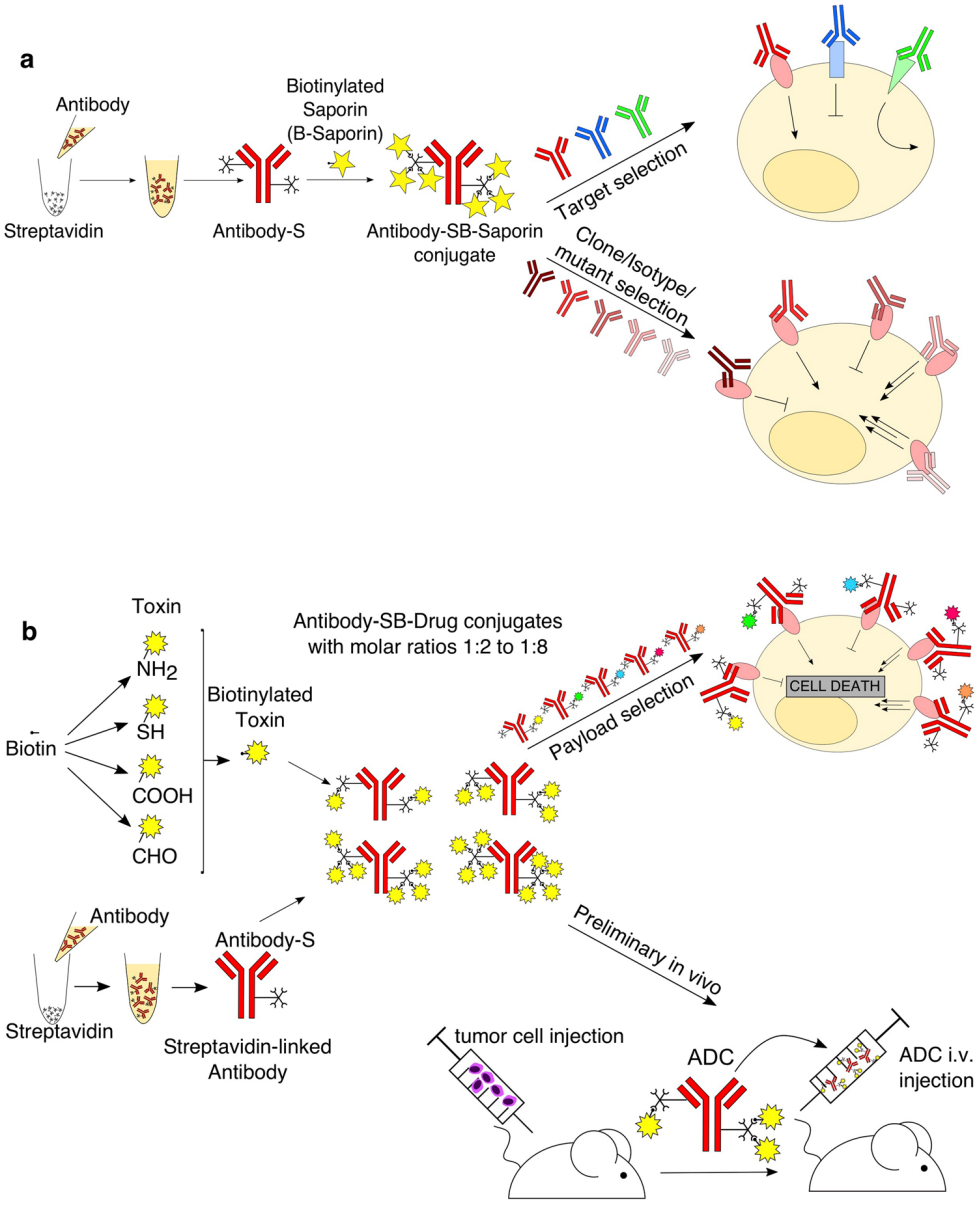
Schematic of applications for the Streptavidin-Biotin linking approach to screen antibodies and payloads for ADC development (**a**) Antibody streptavidin labelling and conjugation to biotinylated Saporin as a quick tool to investigate antibody internalization. This can be applied for target selection of antibodies as well as to screen different antibody clones/isotypes/mutants to a particular target to select for the most suitable antibody for ADC development. (**b**) Antibody streptavidin labelling and conjugation to biotinylated payloads can be used for payload selection and preliminary *in vivo* studies. Commercially available biotinylation kits can be used to biotinylate toxins of interest for conjugation to streptavidin-linked antibodies to generate Antibody-SB-Drug conjugates with different drug-antibody ratios (DARs) due to the tetravalency of Streptavidin. Antibody-SB-Drug conjugates can then be used *in vitro* for payload selection and *in vivo* for preliminary studies.

When choosing a target for ADC development, it is important to differentiate between antigens that do not internalize upon antibody binding, antigens that internalize with the antibody but rapidly recycle to the cell membrane and antigens that get internalized without rapid recycling of the antibody (Fig. 5a)^23^. Only the latter option is a valuable target for ADC development so the antibody can act as a vehicle to specifically deliver payloads to cells expressing the target of interest^24^. To investigate internalization of a range of antibodies we evaluated antibody streptavidin labelling *via* lysines using readily-sourced reagents, and conjugation to biotinylated Saporin for the rapid (*i.e*., under 4 hours) generation of Antibody-SB-Saporin conjugates that can be used for *in vitro* internalization screening. Antibody-SB-Saporin conjugates can reach up to 300 kDa due to the size of Streptavidin and the biotinylated Saporin. However, we showed that Trastuzumab-SB-Saporin could bind Her2, albeit with a decreased affinity compared to naked trastuzumab. This can be due to the steric hindrance of Streptavidin and Saporin or to an impaired capacity of the detection antibody to bind Trastuzumab-SB-Saporin resulting in impaired detection of ADC binding. Furthermore, since in the case of trastuzumab one of the lysine residues potentially conjugated to streptavidin is located in the complementarity-determining region 1 (CDR1) of the heavy chain of trastuzumab (Supplementary Fig. 1a), this could impact the binding of the ADC to Her2. The presence of lysines in the antibody structure including the variable region is therefore a factor to be considered when interpreting findings using this conjugation method. We generated four different Antibody-SB-Saporin conjugates and investigated their internalization profiles in various cell lines with high and low target antigen expression. Saporin-triggered toxic effects, were seen with treatment with ADCs, but not with naked antibodies or payloads alone, and against high tumour antigen-expressing but not low antigen-expressing cancer cells. This suggested that Saporin-based reporter assay is a good method to evaluate antibody internalization, and that internalization of Streptavidin-Biotin linked ADCs, as conventionally-linked ADCs, is target specific.

To easily and rapidly generate Antibody-SB-Drug conjugates (*i.e*., within 7 hours) (Fig. 2a) for *in vitro* and *in vivo* testing, we biotinylated payloads using commercially-available biotinylation reagents and then conjugated them to streptavidin-linked antibodies. As payloads need to be amenable to covalent linking^12^, they must contain chemically-reactive functional groups such as amino, thiols, carboxylic acid or aldehyde groups. Importantly, different biotinylation kits can be purchased to accommodate this range of functional groups. (Fig. 5b). However, payloads that require the presence of their amino, thiol, carboxylic or aldehyde groups in a free form to maintain their cytotoxic potency could not be used, which is a limitation for this platform. The same reactive groups used for biotinylation with our approach could be exploited for commercial ADC generation, rendering early screening with this method readily translatable to subsequent conventional ADC development.

To understand whether a conjugate generated with Streptavidin-Biotin linking could have functional potency similar to a conventionally-linked clinically-applied ADC, we generated an ADC composed of trastuzumab and the emtansine DM1 (Trastuzumab-SB-DM1), and we compared it to clinical-grade T-DM1. Both ADCs exhibited comparable Her2-specific *in vitro* and *in vivo* activity with similar potency profiles. This suggested that Antibody-SB-Drug conjugates could provide an early indication of the suitability of the antibody and payload entity for further study and potential development.

Furthermore, we generated a different Antibody-SB-Drug conjugate based on the MOv18 antibody, targeting the tumour antigen folate receptor alpha, and using the Src-family kinase inhibitor A-419259 as payload. Despite the relatively low potency of the Src-family kinase inhibitor alone compared to the tubulin inhibitor DM1, MOv18-SB-A-419259 was cytotoxic *in vitro* in accord with previously-published results for this ADC^15^. Additionally, the ADCs Trastzumab-SB-DM1 and MOv18-SB-A-419259 were produced by biotinylation through the thiol and primary amine groups of their payloads, respectively. Both thiol and primary amine groups are used in conventional conjugation methods, demonstrating the versatility and applicability of this ADC production methodology to downstream ADC assembly and development.

To our knowledge, this is the first methodology described for the pre-screening of combined antibodies and payloads for future ADC development. Based on readily-sourced reagents for payload biotinylation and antibody streptavidin labelling, and taking advantage of the extremely strong non-covalent interaction between Streptavidin and Biotin^25^, this strategy benefits from rapid (*i.e*., 7 hours) and seamless generation of antibody-drug pairs for *in vitro* and *in vivo* pre-clinical analysis. This conjugation approach is not intended for use as a linking technology for clinical ADC development due to its potential immunogenicity^26,27^, increase in molecular weight^28^, non-covalent linking^27^ and potential interaction of Streptavidin sites with endogenous serum Biotin^29,30^. However, it is noteworthy that the ADCs produced in this study had similar efficacy and toxicity in xenograft models compared to the equivalent ADCs with payloads linked through standard methods. Following evaluation of promising antibody and payload combinations, the optimal DAR could then be investigated for the selected antibody and payload candidates when conjugated via conventional homogenous methods. At early stages of ADC development, molar ratios of biotinylated drug conjugated per antibody could be estimated by using activated biotin labelled with a UV-traceable chromophore for conjugation and then measuring the UV absorbance of the antibody-SB-drug conjugate^31^. The streptavidin/biotin labelling approach described can rapidly elucidate antibody and payload suitability for further ADC evaluation and development prior to investing in conventional conjugation methods (Fig. 5). This should benefit the field by helping to widen the scope of potential antibody and drug combinations to be tested, thus driving translation of more effective ADCs towards clinical application.

## Materials and Methods

### Cell lines

The human breast cancer cell lines HCC1954, SKBR-3, MDA-MB-231, MDA-MB-468 and CAL51 were obtained from King’s College London (KCL) Breast Cancer Now Unit. The melanoma cell lines A375, A2058, SBCL-2 and the monocytic cell line U937 were obtained from the American Type Culture Collection [ATCC] (Manassas, VA). Cell lines were authenticated by short tandem repeat profiling. Cells were used once tested negative for mycoplasma by PCR as described in van Kuppeveld *et al*.^32^. HCC1954, MDA-MB-468 and the human monocytic cell line U937 were cultured in RPMI supplemented with 10% foetal calf serum (FCS); SKBR-3, MDA-MB-231, CAL51, A375, A2058 and SB-CL2 cells were cultured in DMEM supplemented with 10% FCS. All cells were maintained at 37 °C in 5% CO_2_.

### Antibodies

Trastuzumab and trastuzumab emtansine (T-DM1) were sourced from the Guy’s and St Thomas’s Hospitals pharmacy. Anti-CSPG4-IgG1 and MOv18-IgG1 were produced as described in Ilieva et al. and Coney *et al*. respectively^33,34^. Both antibodies were purified using Protein-A columns (Thermo Fisher, 20356) according to the manufacturer’s instructions.

### Antibody-Drug Conjugate (ADC) generation

Antibody-Saporin Conjugation: Antibodies were linked to streptavidin for 3 hours using the Lightning-Link Streptavidin Conjugation Kit (Expedeon, 708-0015) according to the manufacturer’s protocol. Streptavidin-linked antibody was incubated for 30 min with biotinylated Saporin (ATSBio, BT-ZAP) at a molar ratio of 1:6 and Antibody-SB-Saporin conjugates were stored at 4 °C until use.

Trastuzumab-Streptavidin-Biotin-Emtansine (Trastuzumab-SB-DM1) conjugation: trastuzumab conjugated to streptavidin as described above. Mertansine (DM1, MedChemExpress, HY-19792) was biotinylated using the EZ-Link BMCC-Biotin (Thermo Scientific, 21900) according to the manufacturer’s instructions at a molar ratio of 8:1. Biotinylated DM1 was added to trastuzumab-streptavidin with a ratio of 3.5:1 to match the drug to antibody ratio (DAR) 3.5 of the clinically available T-DM1 and the mix was incubated at room temperature for 30 min. Trastuzumab-SB-DM1 was purified using NAb Protein G Spin columns (Thermo Scientific, 89953) and resuspended in PBS for functional experiments.

MOv18-A419259 was produced as previously described (reference Cheung *et al*.), briefly MOv18 IgG1 was linked to streptavidin as described above and A-419259 (Cayman Chemical, 25605) was biotinylated using EZ-Link-Sulfo-NHS-Biotin (Thermo Fisher Scientific, 21425) (molar ratio 8:1). Biotinylated A-419259 and streptavidin-linked MOv18-IgG antibody were incubated at room temperature for 30 min, followed by purification using centrifugation with 3 K Amicon ultra centrifugal filters.

### Investigation of Antibody Fab- and Fc-mediated Binding

Adherent cells were detached with 5 mM EDTA in PBS centrifuged at 500 g for 5 min and resuspended in FACS buffer (2% FBS in PBS). Cells in suspension were centrifuged at 500 g for 5 min and resuspended in FACS buffer. Cells were seeded at 10^5^ cells/100 μl in 5 ml round-bottom polystyrene tubes and treated with different concentrations of antibody or ADC. Cells were incubated for 30 min at 4 °C and then washed with FACS buffer at 500 g for 5 min, followed by a 30 min incubation at 4 °C with 50 μl fluorescein anti-human IgG, diluted in FACS buffer (3:100). Cells were then washed as above, resuspended in 100 μl FACS buffer and analysed on a FACSCanto II flow cytometer.

### Cell viability assays

Adherent cells were seeded at 1000 cells/100 μl per well in 96-well plates 16 hours prior to treatments. Cells in suspension were seeded at the same conditions and treated immediately. Cells were treated with antibody or ADC at final nM concentrations of 10, 1, 0.1, 0.01 and 0.001 or with unconjugated drugs at molar concentrations equivalent to ADC treatment depending on the DAR and incubated for 72-96 hours. Control samples were treated with PBS alone. At the end of incubations, cell supernatants of adherent cells were removed and replaced by normal culture medium supplemented with 10% MTS ([3-(4,5-dimethylthiazol-2-yl)-5-(3-carboxymethoxyphenyl)-2-(4-sulfophenyl)-2H-tetrazolium, inner salt; Promega, G3581). For cells in suspension, 10 μl of MTS was added per well. Cells were incubated for 2 hours in the dark and absorbance was detected on a FLUOstar Omega microplate reader (BMG Labtech) at 490 nm and 650 nm to determine viable cell counts.

### Confocal microscopy

HCC1954 cells were seeded on coverslips at 2×10^4^ cells per well per 24-well plate and treated with 20 μL of 100 nM trastuzumab, T-DM1 and Trastuzumab-SB-DM1 or PBS. After 72 h, cells were washed twice with PBS, fixed with 4% PFA in PBS for 10 min at room temperature. Then, cells were washed with PBS-T (0.1% Triton in PBS) and blocked for 1 hour at room temperature in blocking buffer (5% FBS in PBS-T). Cells were stained overnight at 4 °C with anti-tubulin antibody (Sigma, T8328, 1:500 dilution in blocking buffer). Cells were washed three times in PBS-T and stained with anti-mouse-488 (Abcam, ab150117, 1:200) and anti-IgG-647 (Thermo Fisher Scientific, A-21445, 1:1000) in blocking buffer for 1 hour at room temperature. Cells were washed 3 times in PBS-T and mounted on slides using prolong Gold Antifade Reagent with DAPI (Thermo Fisher Scientific, P35931). Samples were imaged at the Point Scanning Confocal A1R microscope (Nikon Centre, KCL).

### Mouse xenograft studies

All procedures were approved by and performed in accordance with Institutional Committees on Animal Welfare of the UK Home Office in compliance with The Home Office Animals Scientific Procedures Act, 1986. For treatment studies, 3-5-week-old NSG mice were injected in the mammary fat pad with 1.5 ×10^6^ HCC1954 cells pre-mixed with BD Matrigel Matrix (BD Biosciences UK, diluted 1:1 with sterile PBS) in a total volume of 50 μl to establish orthotopic human breast tumour xenografts. On day 9, once tumours were palpable (>4mm^3^), mice received a single intravenous injection of 4 mg/kg trastuzumab, T-DM1, Trastuzumab-SB-DM1 or PBS. Tumours were measured twice per week with callipers and tumour volumes calculated using the formula (π × length × width^2^/6). Mice were weighed when cells were implanted (Day 0) and then twice weekly during the study. Experiments were terminated after 28 days or when tumour sizes measured ≥750 mm^3^.

### Statistical analyses

GraphPad Prism was used for statistical analyses. Data were presented as mean ± standard error of the mean (SEM). Differences with P-values <0.05 were considered statistically significant and all tests were two-sided.

## Conclusions

In this study we have demonstrated conjugation of streptavidin-linked antibodies to biotinylated payloads as a rapid approach to generate Antibody-SB-Drug conjugates for *in vitro* and *in vivo* evaluation. For antibody selection, we showed how Antibody-SB-Saporin conjugates, generated in less than 4 hours, can be used to investigate the antibody’s ability to internalize, a necessity for ADC development. For payload selection we demonstrated that toxins with functional groups normally used for conjugation such as thiols or primary amines can be biotinylated to generate multiple ADCs by conjugation to streptavidin-linked antibodies. We confirmed antitumour activity and explored pre-clinical safety. We generated Trastuzumab-SB-DM1 and compared it to the conventionally-linked and clinically available T-DM1, and we demonstrated equivalent mechanism of action and comparable tumour-growth restricting qualities *in vitro* as well as *in vivo*, in the absence of systemic toxicities. This is a positive step towards facilitating and accelerating the pre-clinical screening of antibody-drug combinations for future ADC development.

## Supplementary information

Supplementary information.
