# Supplementary material for: Rapid conjugation of antibodies to toxins to select candidates for the development of anticancer Antibody-Drug Conjugates (ADCs)
Source: Sci Rep. 2020 Jun 1;10:8869. doi: 10.1038/s41598-020-65860-x (PMC7264231; doi:10.1038/s41598-020-65860-x)
Supplement: Supplementary file 1 — Supplementary information. [file 41598_2020_65860_MOESM1_ESM.pdf]

Rapid conjugation of antibodies to toxins to select candidates for the development of anti-cancer Antibody-Drug Conjugates (ADCs)

Ricarda M Hoffmann[1,2], Silvia Mele[1], Anthony Cheung[1,3], Daniel Larcombe-Young[3], Gintare Bucaite[7,8], Eirini Sachouli[1], Iva Zlatareva[1], Hassan OJ Morad[1], Rebecca Marlow[3,4], James M McDonnell[7,8], Mariangela Figini[9], Katie E Lacy[1], Andrew J N Tutt[3,4], James F Spicer[10], David E Thurston[5,6], Sophia N Karagiannis[1,2,3], Silvia Crescioli[1,2,*]

Supplementary Materials: Fig. S1: Streptavidin-Biotin conjugation of Saporin to trastuzumab can be performed at different molar ratios resulting in decreased cell binding cell viability of ADCs conjugated at higher ratios. Fig. S2: Streptavidin-Biotin conjugation of Saporin to trastuzumab does not potentiate off-target toxicity compared to clinically available T-DM1, Fig. S3: Streptavidin-Biotin conjugation of DM1 to trastuzumab does not potentiate off-target toxicity compared to clinically available T-DM1.

a

Heavy chain trastuzumab

EVQLVESGGGLVQPGGSLRLSCAASGFNI**K**DTYIHWVRQAPG**K**GLEWVARIYPTNGYTRYADSV**K**GRFTISADTS**K**NT
AYLQMNSLRAEDTAVYYCSRWGGDGFYAMDYWGQGTLVTVSSAST**K**GPSVFPLAPSS**K**STSGGTAALGCLV**K**DYFPEP
VTVSWNSGALTSGVHTFPAVLQSSGLYSLSSVVTVPSSSLGTQTYICNVNH**K**PSNT**K**VD**KK**VEP**K**SCD**K**THTCPPCPA
PELLGGPSVFLFPP**K**P**K**DTLMISRTPEVTCVVVDVSHEDPEV**K**FNWYVDGVEVHNA**K**T**K**PREEQYNSTYRVVSVLTVL
HQDWLNG**K**EY**K**C**K**VSN**K**ALPAPIE**K**TIS**K**A**K**GQPREPQVYTLPPSRDELT**K**NQVSLTCLV**K**GFYPSDIAVEWESNGQP
ENNY**K**TTPPVLDSDGSFFLYS**K**LTVD**K**SRWQQGNVFSCSVMHEALHNHYTQ**K**SLSLSPG**K**

Light chain trastuzumab

DIQMTQSPSSLSASVGDRVTITCRASQDVNTAVAWYQQ**K**PG**K**AP**K**LLIYSASFLYSGVPSRFSGSRSGTDFTLTISSL
QPEDFATYYCQQHYTTPPTFGQGT**K**VEIRTVAAPSVFIFPPSDEQL**K**SGTASVVCLLNNFYPREA**K**VQW**K**VDNALQSG
NSQESVTEQDS**K**DSTYSLSSTLTLS**K**ADYE**K**H**K**VYACEVTHQGLSSPVT**K**SFNRGEC

V_H and V_L: Framework regions (FRs), Complementarity-determining regions (CDRs)
C_H and C_L: Constant region
mAb: **Lysines (K)**

b

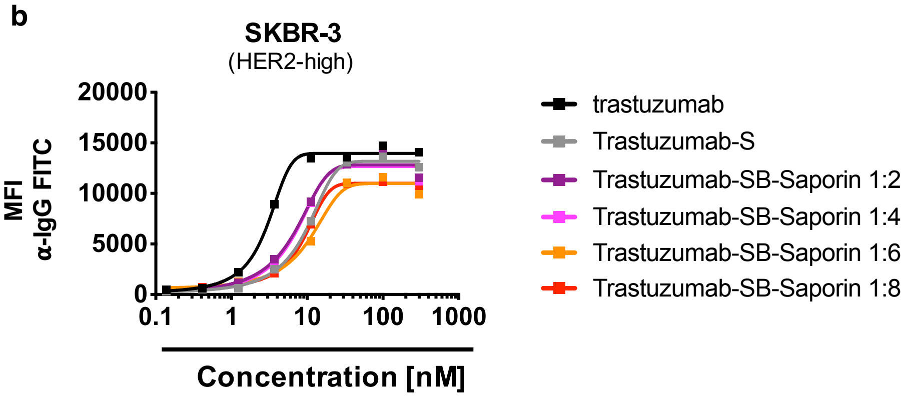

c

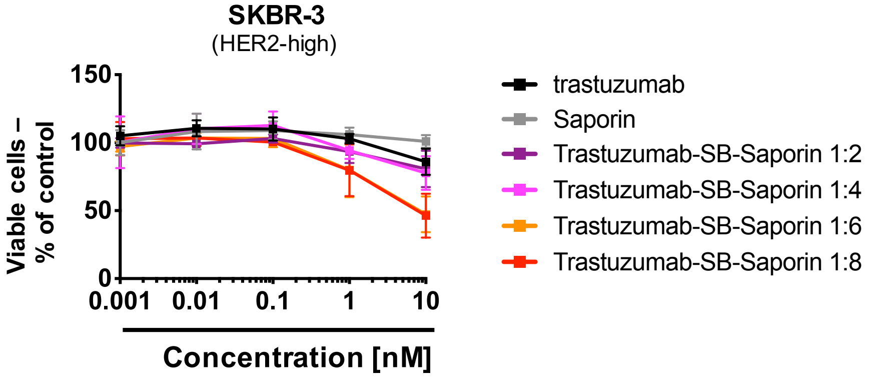

Supplementary Fig. 1. Streptavidin-Biotin conjugation of Saporin to trastuzumab can be performed at different molar ratios resulting in decreased cell binding and cell viability of ADCs conjugated at higher ratios. (**a**) Sequence of heavy and light chain of trastuzumab highlighting lysine (K) residues available for conjugation to Streptavidin in framework regions, complementarity-determining regions and constant regions. Streptavidin labelling by using the LightningLink Streptavidin kit results in the conjugation of an average of 2 molecules of Streptavidin per antibody at 41 possible lysine residues. (**b**) Investigation of cell binding of breast cell line SKBR-3 with high Her2 expression of Trastuzumab-SB-Saporin ADCs conjugated with 4 different ratios from 1:2 to 1:8. Streptavidin-linked trastuzumab shows reduced binding affinity to naked trastuzumab. (**c**) Investigation of cell viability of breast cell line SKBR-3 with high Her2 expression of Trastuzumab-SB-Saporin ADCs conjugated with 4 different ratios from 1:2 to 1:8 showing reduction in cell viability for ADCs with more Saporin conjugated per antibody. N=1 for all binding assays and N=3 independent experiments for all MTS studies, each condition performed in triplicate, error bars represent Standard Deviation (SD).

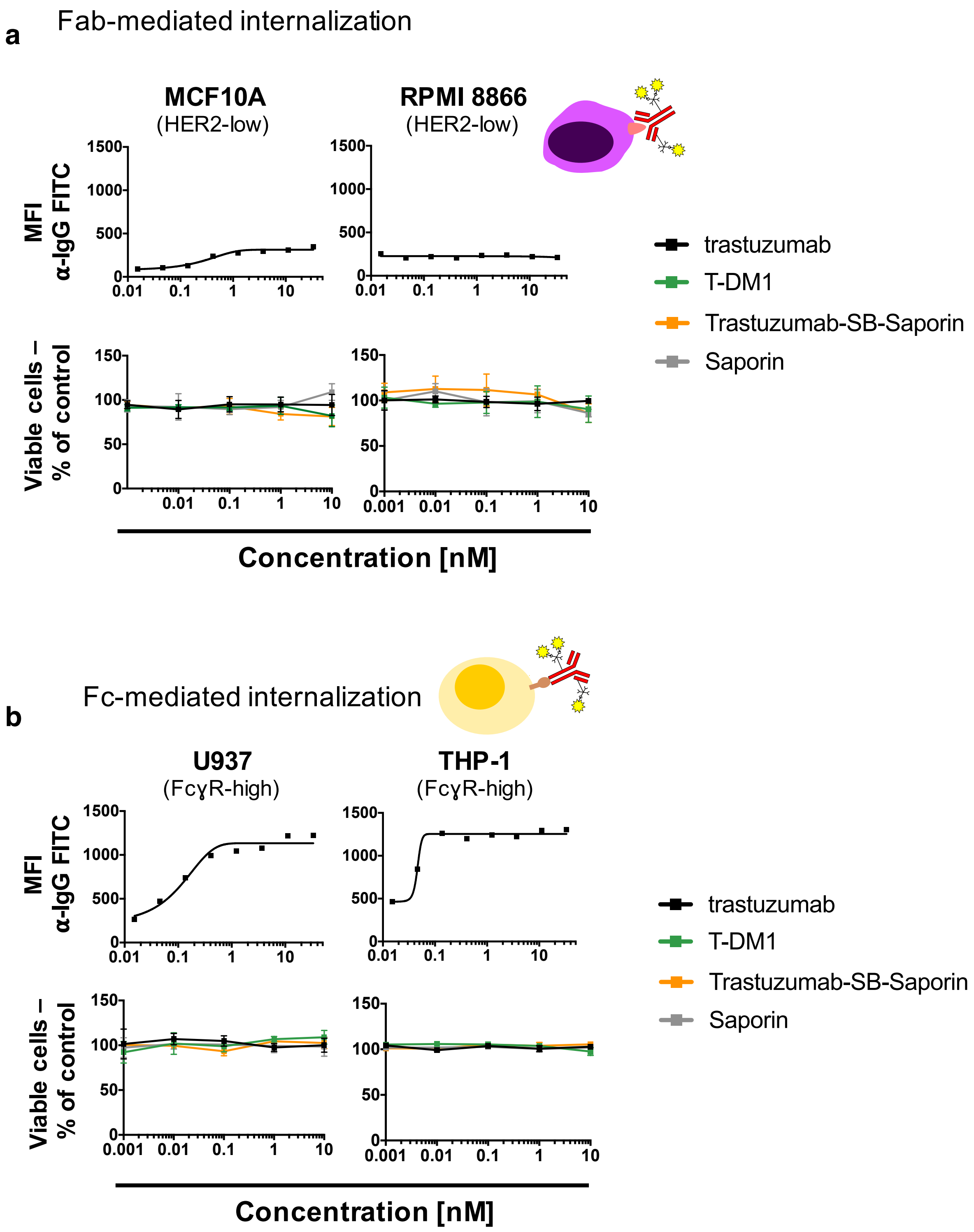

Supplementary Fig. 2. Streptavidin-Biotin conjugation of Saporin to trastuzumab does not potentiate off-target toxicity compared to clinically available T-DM. (**a**) Investigation of cell viability of breast cell line MCF10A and myeloid cell line RPMI 8866 with low Her2 expression. (**b**) Investigation of cell viability of monocytic cell lines U937 and THP-1 with high Fcγ-receptor expression. Cells were treated with the naked antibody trastuzumab, control ADC T-DM1, Trastuzumab-SB-Saporin or Saporin toxin alone. N=1 for all binding assays and N=3 independent experiments for all MTS studies, each condition performed in triplicate, error bars represent Standard Deviation (SD).

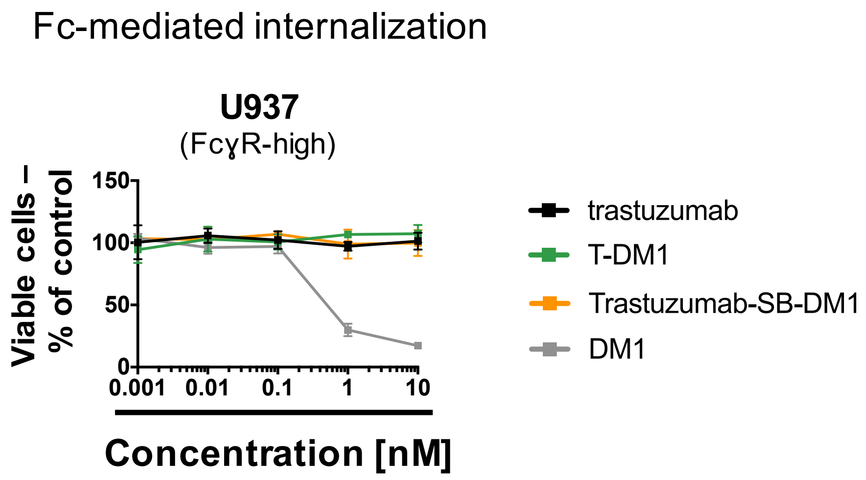

Supplementary Fig. 3. Streptavidin-Biotin conjugation of DM1 to trastuzumab does not potentiate off-target toxicity compared to clinically available T-DM1. Investigation of cell viability of monocytic cell line U937 with high Fcγ-receptor expression. Cells were treated with the naked antibody trastuzumab, control ADC T-DM1, Trastuzumab-SB-DM1 or DM1 toxin alone. N=3 independent experiments, each condition performed in triplicate, error bars represent Standard Deviation (SD).
